# Clinical features, evaluation, and management of speech and swallow complications of facial paralysis: A scoping review

**DOI:** 10.1016/j.jpra.2025.10.013

**Published:** 2025-10-20

**Authors:** Mia Vargo, Peng Ding, Michelle E. Adessa, Radhika Duggal, Ravi Dhamija, Yiqing Tang, Trisha Shang, Katherine Wang, Mary Schleicher, Dane J. Genther, Patrick J. Byrne

**Affiliations:** a School of Medicine, Case Western Reserve University, Cleveland, OH, USA; b Otolaryngology - Head and Neck Surgery, Cleveland Clinic, Cleveland, OH, USA; c Cleveland Clinic Lerner College of Medicine, Cleveland Clinic, Cleveland, OH, USA; d Department of Neuroscience, Ohio State University, Columbus, OH, USA; e Cleveland Clinic Floyd D. Loop Alumni Library, Cleveland, OH, USA; f Department of Cognitive Science, Case Western Reserve University, Cleveland, OH, USA

**Keywords:** Facial paralysis, Oral competence, Speech complications, Assessment tools, Treatment methods, Literature review

## Abstract

**Objective:**

Facial paralysis may lead to complications such as dysarthria, oral residue during deglutition, dysphagia, and sialorrhea. Literature regarding the assessment and treatment of these complications in patients with facial paralysis has historically been limited. However, there continues to be new evidence in this field that aims to fill the gaps in our current understanding of this condition and its implications for patients. The primary aim of this study was to analyze the current literature regarding the effect of facial paralysis on speech and oral competence, discuss treatment options, and assess implications for the future.

**Data Sources:**

Ovid® Medline ALL, Ovid Embase, and Cochrane Library from Wiley.

**Review methods:**

A scoping review of the literature was conducted to investigate the effects of facial paralysis on speech and oral competence, as well as current evaluation and treatment methods in adult populations. Studies were assessed based on set inclusion criteria relevant to the goals of the review.

**Conclusion:**

Facial paralysis may lead to various speech and swallowing challenges. Numerous assessment tools and treatment options exist for facial paralysis, however, evidence regarding their effectiveness in managing oral complications is limited.

**Implications for practice:**

There is a need for consideration as well as standardization of the effect of facial paralysis on speech, swallowing, and saliva management. It may be reasonable to consider the inclusion of a speech language pathologist on the care team allowing for the early assessment of oral complications related to communication and deglutition. Additionally, there is need for both subjective and objective evaluation methods throughout the care timeline. Utilizing a broader interdisciplinary approach to management could potentially improve both patient outcomes and experience.

## Introduction

Facial paralysis (FP) also known as facial nerve paralysis (FNP) is a condition characterized by the paralysis of structures innervated by the facial nerve.^1^ Common causes include idiopathic palsy, trauma, infections, and congenital factors.^2^ Physiological complications are frequently observed, such as facial weakness,^3^ exposure keratitis,^3^ and difficulty expressing emotions.^4^ These challenges significantly impact patients’ daily lives, often leading to psychological complications like depression,^5^ negative social perceptions,^6^ and a reduced quality of life (QOL).^7^ The oral region plays a critical role in essential functions such as speech and swallowing, both of which can be severely affected by FP.^1^ The literature has historically overlooked the impact of FP on oral dysfunction, including its effects on deglutition, speech, and saliva management. However, new research continues to fill the gaps in our current understanding of this condition and its implications for patients. Charters and Coulson previously conducted a systematic review and quality analysis of oral competence outcomes following facial paralysis.^8^ Their review emphasizes methodological rigor and quality analysis of their included studies, providing insight into how this topic can be researched more effectively. In contrast, our scoping review intentionally incorporates a broader range of study designs to assess the clinical manifestations, assessment strategies, and treatment options reported in the current literature. Additionally, 10 of our included studies were published following the publication of their review. By employing more inclusive criteria, our goal is to provide a comprehensive review of the literature for clinical applications, offering a practical synthesis of available evidence for healthcare providers that address challenges in managing speech and swallowing outcomes in patients with FP.

## Methods

**Figure 1:**
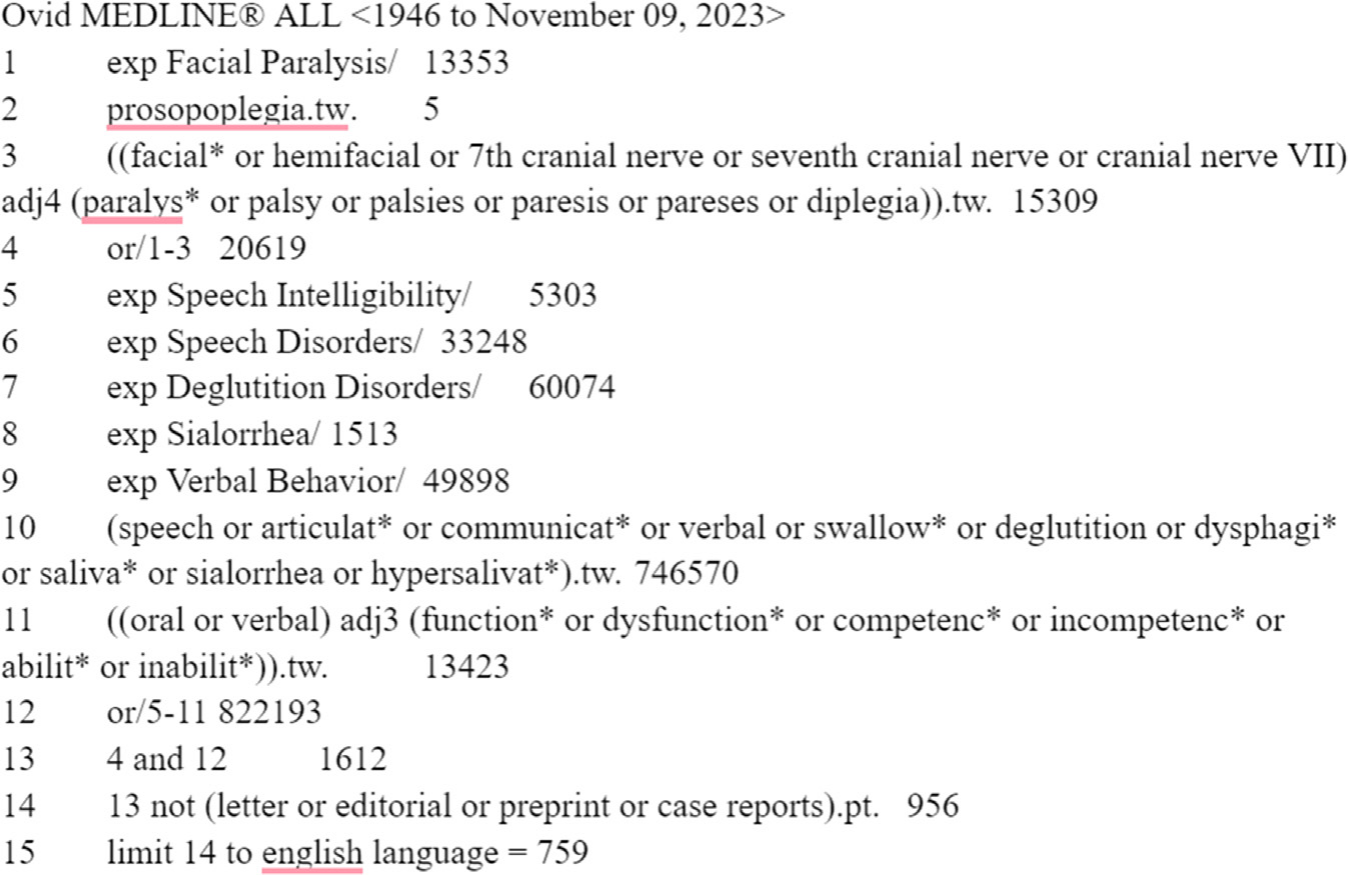
Total search strategies.

**Figure 2:**
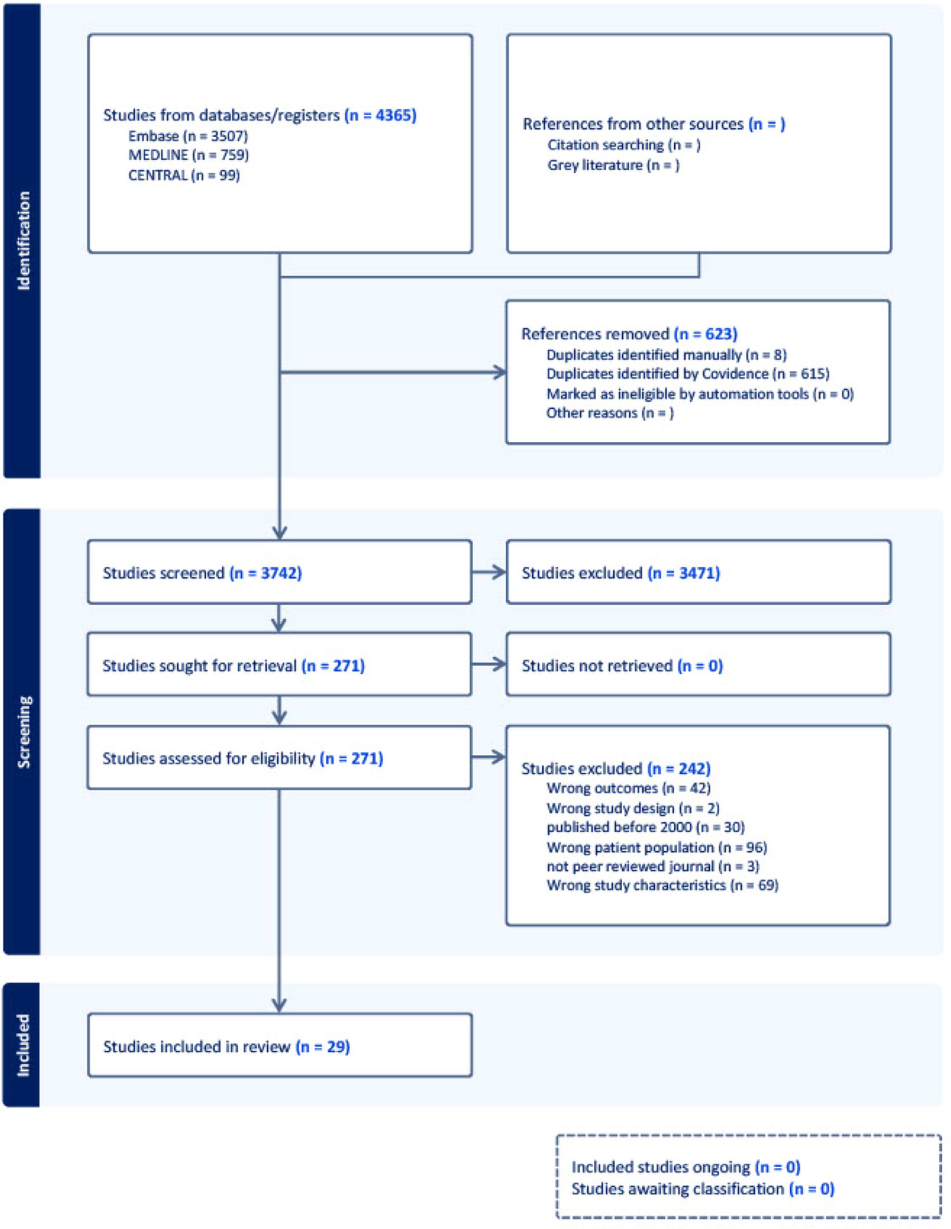
Preferred reporting items for systematic reviews and meta-analyses (PRISMA) flow diagram.

A comprehensive search was created to combine the concepts of facial paralysis or seventh cranial nerve paresis with terms for speech and swallowing disorders, excessive salivation, and verbal, oral or communication dysfunction. Indexing terms and text words were used for all concepts, utilizing truncation and adjacency operators to increase the retrieval of potentially relevant material. All searches were run from database inception and publication types for letters, editorials, case reports and conference abstracts were removed where those filters existed. Results were limited to the English language only. The following databases were searched on November 10, 2023: Ovid® Medline ALL, Ovid Embase, and Cochrane Library from Wiley. by our librarian. A title and abstract screening was conducted by authors followed by a full text screening by authors one through eight (MV, PD, MA, DR, RD, YT, TS, KW). Final selection and inclusion of articles was determined by the first (MV) and second (PD) authors. Inclusion and exclusion criteria are outlined in Table 1. Once the list of included studies was confirmed, data was extracted and assessed by the authors who conducted the full text screening. The Medline strategy is outlined in Figure 1. Search and screening results are described in Figure 2. Given that the purpose of our scoping review is to encompass a wide breadth of topics and study designs, a formal quality analysis was not performed to ensure the inclusion of all relevant literature.

**Table 1:** Inclusion and exclusion criteria.

	Include	Exclude
Population	Adults Peripheral facial paralysis/palsy Patients with oral incompetency—speech or swallowing Speech: articulation disorder, slurred speech Swallowing: leakage, oral incompetency, food containment, biting cheeks, biting lips	Central FP
Outcome	Clinical features of speech or swallow: incidence Evaluation of speech or swallowing Management of speech or swallowing: speech therapy, rehabilitation, facial retraining, physical therapy, surgery	Etiology only Post-stroke dysphagia or dysphonia Validation of facial grading system, comparing with commonly used system or in different language
Study characteristics	Clinical study: Human only Published after 2000	Case report Case study Clinical trial protocol Animal study Published before 2000

## Results

A total of 4365 citations were uploaded into Covidence and 623 duplicates were identified manually or by the software, leaving 3742 citations at the title & abstract screening stage. Reviewers assessed 271 full-text studies for eligibility. A final set of 29 studies were included.

The most represented study type was a cohort study, with 26 studies (90 %), followed by two randomized controlled trials (7 %), and one case series (3 %). Among all 29 studies, 15 (52 %) discussed both speech and swallowing dysfunction. 13 (45 %) studies focused only on one dysfunction: six studies (21 %) focused on issues regarding eating, drinking, and swallowing only, while seven studies (24 %) addressed speech independent of swallowing function. The other one study (3 %) focused on a topic related to facial dysfunction or treatment caused by FP or assessed areas that were indirectly related to oral competence.

### Complications and clinical features

#### Dysphagia

Abbas-Kayano et al.^9^ found evidence that cranial nerve palsy following acoustic neuroma surgery has long-term (>2 years) effects on dysphagia. Their study employed a structural assessment of the oral phase of swallowing and the Functional Oral Intake Scale (FOIS). They found that patients with FP had worsened facial function (55 %), dysphagia (46 %), and challenges with thin liquid intake (80 %). All dysphagic individuals reported significant impairment in daily activities related to the condition.

Venables et al.^10^ found similar results in 26 patients with lower motor neuron facial palsy. Dysphagia and difficulty controlling a bolus in the mouth were the most prevalent issues in their cohort, which they attributed to possible poor innervation of the affected orbicularis oris and buccinator muscles. Movérare et al.^11^ found that difficulties with eating and drinking in were most commonly related to food and drink leaking out of the corner of the mouth (70 %) and residue in the mouth after meals (59 %) in 27 patients with FP, and that these patients also exhibited lower lip force when measured with a lip force meter. Montino et al. had similar reports from their participations with FP, with self-reported complaints of liquid dribbling out of the lips and difficulty locating food inside the mouth which impacted oral clearance.^12^

Seçil et al.^13^ compared swallowing dysfunction in 44 patients with Bell’s palsy to that of controls. They found that patients with FP had increased difficulty controlling a bolus in the mouth (79 %) and an increased incidence of dysphagia (55 %). In patients with FP, Kato et al.^14^ saw impaired masticatory efficiency and oral vestibular cleansing capability associated with facial nerve paralysis as well. Both Seçil et al. and Kato et al. noted that patients who exhibited recovery from their FP found improvements with their deficits, further suggesting the existence of a connection between FP and the oral processes of eating and drinking.

#### Communication

Movérare et al.^11^ used questionnaires, percentage of words correctly understood (PWC), percent of consonants correct (PCC) and clinical assessment by a speech language pathologist (SLP) to assess speech in patients with FP versus reference data. They found a statistically significant difference in articulation between FP patients and controls, particularly in labial consonants such as weak pressure articulation of /f/, and bilabial consonants /b/ and /p/. However, these errors did not severely impact intelligibility. Severity of FP did not directly correlate with articulation deficits, but there was a significant link between articulation, intelligibility, and self-perceived communication ability, indicating that FP severity as graded by scales such as the Sunnybrook Facial Grading Scale (SFGS) may not provide an overall picture of the difficulties caused by facial paralysis. Charters et al.^15^ analyzed the speech of 40 patients with FNP compared to controls using the Speech Handicap Index (SHI) and perceived intelligibility as rated by an SLP, community member, themselves, and diction software. They noted lower intelligibility in patients with FP than controls, particularly bilabial sounds (consonants: /b/, /p/ and /m/) and lingual fricatives requiring lip retraction (/s/, /z/, /sh/). The participants with FP also rated their intelligibility worse than when assessed by a professional. Hayler et al.^16^ reported similar findings in 40 patients with FNP, whose speech was evaluated using the Facial Disability Index (FDI), SHI, and intelligibility scores assessed by an SLP, a member of the public, and the patient. They also noted that females with FP rated their intelligibility lower than males. Kim et al.^17^ conducted a nationwide online survey of 160 adults with unilateral FP using the Communicative Participation Item Bank (CPIB) Short Form questionnaire and the Facial Clinimetric Evaluation (FaCE) Scale. They found that patients with FP in their study reported restrictions in communicative participation that were comparable with restrictions experienced by patients with other known communicative disorders, such as laryngectomy and head and neck cancer.

#### Additional considerations

Movérare et al.^11^ found that 46 % of participants with FP self-reported difficulties controlling saliva, mainly experiencing mild to moderate drooling on the lips and chin. However, only 15 % reported that drooling was a problem. Additionally, the patients who perceived it as a problem did not have a direct relationship with the amount of drooling. Thus, objective assessment of sialorrhea was not sufficient in predicting patient distress.

Subramaniam et al.^18^ used questionnaires to measure QOL in patients who underwent different types of parotidectomies. Patients that underwent a radical parotidectomy rated their speech and taste abilities significantly worse than patients who underwent a limited superficial parotidectomy. They were also found to have significantly higher levels of dissatisfaction with their appearance.

### Assessment methodologies

Questionnaires were the primary method for assessing oral function. Identified questionnaires were the FOIS, SHI, Oral Competence Questionnaire (OCQ), Visual Analog Scales (VAS), University of Washington Quality of Life Questionnaire, Parotidectomy Specific Quality of Life Questionnaire, CPIB Short Form, Synkinesis Assessment Questionnaire (SAQ), Inventory of Patient-Reported Eating and Drinking Problems (IPREDD) as well as several others, many of which were created by the authors for the purposes of their study. Self-reported speech and oral competence and deficits were also included.

#### Objective oral measurements

The Iowa Oral Performance Instrument (IOPI), was used to assess facial function objectively by quantifying muscle strength in the lips and tongue in a study by Starmer et al.^19^ Analysis of speech recordings by patients with FP were also utilized to calculate measures such as the PCC.^15^ Kato et al.^14^ assessed mastication efficiency by looking visually in the oral cavity following chewing as well as measuring glucose eluted from a jelly candy after chewing.

#### Role of speech pathology assessment for patients with FP

Evaluation and clinical assessments by SLPs or clinicians were used routinely to assess oral competence and speech. These were typically used in conjunction with validated FP classification systems, such as the SFGS and House-Brackmann (HB) score. Multiple studies emphasized the importance of speech language pathologists on the care team.^9^^,^^11^^,^^12^ Speech language pathologists were frequently involved in analyzing speech for intelligibility and articulation deficits, completing comprehensive swallowing assessments, or administering treatment for patients with FP.^11^^,^^16^^,^^17^^,^^20^^,^^22^^,^^23^

## Treatment

### Non-surgical treatment

#### Facial retraining

Beurskens and Heymans found significant improvements in facial stiffness and lip mobility in FP patients after 10 weeks of mime therapy.^24^ They also conducted a retrospective review of 155 patients with peripheral FNP who received 3–6 months of mime physiotherapy and found that patients reported significant improvement in impairment, disability, and QOL regarding eating, drinking, and speaking when assessed using self-reported scales.^25^ Venables et al.^10^ found that neuromuscular rehabilitation was shown to improve oromotor function and social engagement in patients with FP using the IPREDD. They found that IPREDD-focused symptoms reduced from 74 % to 43 %. Martineau et al.^26^ conducted a randomized controlled trial to compare the effects of basic therapy counseling versus a “Mirror Effect Plus Protocol” (MEPP) in Bell’s palsy patients. MEPP included motor imagery, manipulations, and facial mirror therapy. They MEPP positively affected House-Brackmann (HB) scores, synkinesis, and QOL. However, when rated by naïve judges before and after therapy, speech intelligibility improvement was similar to controls.^26^

### Office based minor procedures

#### Lip injection augmentation

Starmer et al.^19^ assessed lip pressure, anterior bolus spillage, and articulation of bilabial sounds before and after targeted lip augmentation with hyaluronic acid (HA). Lip pressure was measured using the IOPI. Following treatment, they found that labial strength improved across the entire lip, and the SLP assessing the patients noted overall improvement in labial strength, articulation of plosive sounds, and bolus spillage without complications.

#### Barbed threads

Costan et al.^27^ used barbed threads for static facial reanimation in FP patients using polydioxanone (PDO) threads and observed improvements in facial appearance, oral competence, and diction post-procedure.

### Botulinum toxin injections

Wamkpah et al.^21^ assessed speech and communication changes after 4–6 weeks following chemodenervation using the CPIB Short Form, assessment by an SLP, and self-reported experiences. They found that the CPIB and Word Intelligibility Test were not significantly different following chemodenervation. Additionally, 50 % reported no change in communication ability.

### Facial reanimation surgery

#### Nerve transfer

Hypoglossofacial anastomosis surgery has been found to be successful in terms of FP severity, but complications such as dysphagia, hemiglossal atrophy, dysarthria, disarticulation, and tongue numbness occurred following hypoglossofacial anastomosis surgery.^28^^,^^29^

#### Static procedures

Charters and Low assessed speech and swallowing in 19 patients with FNP before and after static sling reconstruction. Their patients demonstrated enhanced oral competency and speech intelligibility due to improvements in bilabial sounds, lingual fricatives, and labiodental sounds.^23^ Autogenous fascia lata grafts were also shown to improve speech, oral competence, fluid retention, and chewing in studies by Lemound et al. and Rose.^30^^,^^31^ A novel method of static midlife suspension using a modiolar rotational cheiloplasty with alar base transposition and gingivobuccal sulcopalsty was shown to improve drooling, buccal stasis of food, dysarthria, and overall appearance.^32^

#### Synkinesis surgery

Miller and Hadlock^33^ conducted a retrospective review on 19 patients with selective denervation surgery for FP. They showed significant improvements in smile, FaCE scores, and discomfort, but 41 % had worse drooling and/or chewing after surgery. New or exacerbated oral incompetence and articulation difficulties arose in four patients (21 %) following the procedure as well. However, Hussain et al.^34^ found no adverse changes in oral continence or speech following a depressor labii inferioris resection.

## Discussion

### Clinical manifestations

Individuals with FP are at an increased risk of dysphagia, as well as difficulties with oral clearance and mastication efficiency. Controlling a bolus in the mouth was one of the most common complaints noted in our review regarding eating, and it has been suggested that reduced tactile sensation in the mouth may contribute to difficulties in locating food.^12^^,^^13^ However, more research into this connection is warranted. Dysphagia can significantly impact a patient’s QOL, including poor mealtime experiences, reduced social engagement, and reduced choice and control.^35^ Therefore, patients may feel embarrassment when eating in public or with others, which can isolate that patient from others. Assessment of dysphagia in patients with facial paralysis is recommended to screen for difficulties.

Articulation and intelligibility can both be impacted by FP, particularly bilabial consonants /b/, /p/, and /m/. While Movérare et al.^11^ saw that these articulation deficits did not impact communication, patients with FP were more likely to rate their intelligibility lower than when assessed by a professional. Previous studies have shown that FP severity doesn’t consistently relate to psychological distress,^36^, ^37^, ^38^ or anxiety and depression,^39^^,^^40^ which may similarly affect perceived intelligibility. Communication greatly impacts QOL^41^^,^^42^ and negative speaking experiences have shown to lead to lower QOL.^41^^,^^43^ If a patient thinks that they will not be understood, they may become discouraged from speaking, ultimately reducing communication participation.^44^

Beyond speech, other oral complications can further influence communication and social participation. Movérare et al.^11^ found increased rates of patients experiencing difficulty controlling saliva, although most of them did not find it to be a problem, and the amount of drool did not correlate with those who found it to be a problem. Therefore, solely objective or subjective measures of drooling may not be sufficient for patients with FP. A holistic approach to speech assessment in patients with FP may prevent lower QOL due to psychosocial factors beyond their objective presentation.

Facial paralysis may be due to a wide range of etiologies, leading to a wide variety of severity levels and differences in presentation between individuals. Based on an article by Subramaniam et al.,^18^ anticipating patients’ expectations and counseling them appropriately improves their ability to cope with the disorder. Understanding the nature of the FP onset is crucial for addressing patient needs and underscores the importance of discussing surgery risks preoperatively. However, due to the nature of FP, most studies incorporate a range of FP etiologies and still have low participant counts. Therefore, it is difficult to evaluate differences in speech and swallow outcomes between etiologies.

Gender also influences the experience of patients with FP. Women with FP tend to report higher levels of anxiety,^38^ depression,^7^ and psychological distress^6^^,^^38^^,^^45^ compared to men. Research has shown that female patients with FP are more critical of their speech and perceive a greater impact of their condition on their lives despite similar clinical scores to males.^43^ This gender difference has implications for both clinical management and research design. For instance, studies with a disproportionate amount of one gender may not fully represent the experience of all participants of different genders. For example, one study examining speech participation was 91 % female.^44^

Taken together, these findings highlight the complex interplay between etiology severity, gender, and patient perceptions in shaping outcomes for individuals with FP. Communication difficulties and oral complications not only affect physical functioning but also psychological well-being and social participation. A comprehensive and patient-centered approach incorporating both objective and subjective assessments and consideration of psychosocial factors are essential for improving patient outcomes.

### Assessment methodologies for those with FP

Questionnaires were the most common assessment method for speech and swallowing complications. However, variability in questionnaire usage and grading scales across studies raises validity concerns. Inconsistency in grading scales across institutions complicates result comparison. Therefore, there is a need for more standardized reporting of functional outcomes like speech and swallowing in patients with FP. Pressure tests, such as the IOPI, assess facial function objectively by quantifying muscle strength in the lips and tongue. Despite the advantages of objective measuring tools, quantitatively measuring facial function with pressure tests is not routinely practiced in FP clinical assessment or research.

Speech language pathologists are essential for evaluating speech and swallowing disorders. They offer important services in the assessment, diagnosis and treatment of communication and swallowing impairments. To effectively address challenges related to oral functioning in patients with FP, it is essential to leverage the specialized skills of healthcare team members trained in diagnosing and treating oral functioning disorders, deglutition and swallowing. Clinical SLP assessment was one of the most common methods our included studies used to measure speech and oral competence.

As there are no current recommendations for speech and swallow assessment for FP, it is recommended to follow well-established speech and swallowing assessments which are completed by SLPs to assess other disorders. This includes a cranial nerve assessment followed by a clinical swallow examination and possible instrumental evaluation with modified barium swallow (MBS) or nasendoscopy, or fiberoptic/flexible endoscopic evaluation of swallowing (FEES), as indicated. Lip strength and endurance may be measured with the Iowa Oral Performance Instrument (IOPI). Useful validated tools to include in assessment may be the Mann Assessment of Swallowing Ability (MASA), the Test of Masticating and Swallowing Solids (TOMASS), and/or the Assessment of Intelligibility of Dysarthric Speech (AIDS). It may be warranted to repeat these evaluations at different points in recovery to assess progress and refine treatment for patients. Furthermore, collecting patient reported outcome measure may also be useful, including the Oral Health Impact Profile (OHIP-14), the Voice Handicap Index (VHI) or Speech Handicap Index (SHI).

### Treatment

Facial retraining, including mime therapy and neuromuscular rehabilitation, show promise as noninvasive options for improving facial movement and function in patients with FP. The findings from our study support the effectiveness of physiotherapy and neuromuscular rehabilitation as noninvasive treatment options for patients. While research has explored the impact of mirror therapy on overall function and quality of life, studies on its influence on speech or swallowing issues are limited.^46^^,^^47^

Minimally invasive interventions such as HA fillers and barbed threads may provide additional therapeutic options for patients with FP. HA fillers, while originally developed for aesthetics, have been shown to be effective in improving lip opening pressure, reducing bolus spillage, and enhancing bilabial sound articulation.^19^^,^^48^ While effective and minimally invasive, improvements are temporary and subsequent injection may be necessary. Barbed threads offer a minimally invasive option to address facial drooping and the inability to smile or express facial emotions. The method is attractive due to its minimally invasive nature and lower cost than traditional facelifts, but has not been used as a treatment for FP until recently. While promising, this approach carries risks such as asymmetry, visibility of sutures, numbness, or a lack of improvement.^49^ Therefore, barbed threads may be a promising alternative to facial reanimation surgery, but more research is needed to assess its efficacy and refine techniques for optimal results in FP treatment.

Botulinum toxin injections are a standard minimally invasive treatment for FP, targeting unwanted movement in the affected side while reducing hyperactivity in the unaffected side, helping restore commissure and lower lip symmetry and oral function.^50^ However, Wamkpah et al.^21^ found no change in speech outcomes following botulinum toxin injection. A study by Pourmomeny et al.^51^ found that botulinum toxin in combination with neuromuscular retraining showed significant improvement in synkinesis and symmetrical improvements compared to botulinum toxin therapy alone. However, this has not been assessed in the context of speech and oral competence measures.

Nerve transfers redirect axons from a functional donor nerve to innervate and restore function in a recipient nerve. Although no donor nerve can guarantee complete recovery, hypoglossal-facial nerve transfer restores resting facial tone and voluntary smile with neuromuscular training.^52^ Techniques like the side-to-end transfer as opposed to the traditional end-to-end transfer aim to reduce these oral complications, and have been shown to be effective.^53^^,^^54^

Static interventions like fascia lata suspension address FP by restoring perioral positioning and function in the flaccid face,^50^ and were found to improve speech and swallow outcomes in FP.^30^^,^^31^ Selective denervation and neurectomy procedures are treatments to alleviate these issues, but can carry risks such as oral incompetence, drooling, and articulation impairment.^48^^,^^55^ Conflicting results from our search suggest further research is needed for conclusive findings.

Together, these findings highlight the range of therapeutic options for FP, including conservative and surgical management. While multiple interventions showed promise for improving oral competence and communication, there is a need for larger, high-quality studies to guide clinical decision-making.

### Study limitations

Research on facial paralysis is notably limited, especially concerning its impact on speech and swallowing. There was a scarcity of studies per topic, with an overrepresentation of cohort and single-institution studies. Sample sizes were typically small, with a heterogeneous mixture of etiologies. Few studies described innovative research methods as well. The primary method for assessing oral competence was subjective questionnaires, some of which were customized by the authors for their study, leading to varied assessment approaches. Additionally, our search lacked a double-checking process during study selection, potentially introducing bias based on individual screening decisions.

The findings of our research align with those of Charters and Coulson, whose review examined 44 articles and found similar limitations to the current research on oral competence and FP.^8^ They also saw an overrepresentation of cohort studies, as well as a lack of validated outcome measures. These implications highlight the importance this search in identifying the limitations and gaps in existing research on FP.

### Implications for practice

Facial paralysis has been shown to significantly affect oral competency, which in turn influences quality of life. Yet, research addressing oral complications, their assessment, and treatment options for FP patients is scarce. While smile restoration is a primary focus in facial reanimation, emerging attention is being directed towards speech and swallowing functions. However, significant gaps persist. More comprehensive research is needed to understand how FP impacts oral functions like bolus control, dysphagia, articulation, and intelligibility. Current studies often rely on unstandardized subjective methods. Standardizing assessment methods across studies is crucial for accurate comparisons, particularly in evaluating treatment efficacy. Objective measures, like pressure tests, should be integrated for more precise assessments. Involving speech-language pathologists in patient care and assessment is also valuable, as they can provide professional assessment and intervention, address issues early and improving clinical outcomes.

It is also important to note that increased severity of FP does not always result in poorer oral functioning. For instance, de Swart et al.^20^ studied 17 patients with unilateral FP and found that nearly all of them experienced difficulties with eating and drinking. However, the objective severity of their FP as graded by a clinician did not correlate with these difficulties. This demonstrates how objective impairment alone does not predict specific challenges or barriers a patient may experience. Therefore, combining both subjective and objective assessment will lead to more holistic patient assessment and improved treatment planning. Furthermore, patients should be informed about potential complications such as FP before undergoing procedures, enabling better adaptation and psychological support. Early intervention is critical; as prompt treatment post-onset has been shown to lead to better outcomes.

## Conclusion

Our study aimed to review current literature concerning speech and swallowing function in the FP population via a scoping review. FP can lead to various oral complications, including reduced lip force, difficulty controlling food, lower mastication efficiency, compromised oral clearance, dysphagia, and drooling. Speech challenges involve reduced articulation, intelligibility, and perceived communication. Influential factors include etiology, severity, and gender. Numerous assessment tools and treatment options exist for FP. However, evidence regarding their effectiveness in managing oral complications is limited. There is a need for standardization and further research in this area. Recommendations include early assessment of oral complications, the inclusion of a speech pathologist on the care team, and a mixture of subjective and objective evaluation to improve patient outcomes.

## Funding

None.

## Ethical approval

None required.

## Conflicts of interest

None.
